# Qualitative lysine crotonylation and 2-hydroxyisobutyrylation analysis in the ovarian tissue proteome of piglets

**DOI:** 10.3389/fcell.2023.1176212

**Published:** 2023-05-15

**Authors:** Diqi Yang, Xiaoping Li, Beibei Yu, Hui Peng

**Affiliations:** School of Animal Science and Technology, Hainan University, Haikou, China

**Keywords:** crotonylation, 2-hydroxyisobutyrylation, posttranslational modification, ovary, piglets

## Abstract

Ovarian function influences diverse aspects of fertility and reproductive lifespan by regulating oocyte supply and hormone secretion. Lysine crotonylation (Kcr) and lysine 2-hydroxyisobutyryllysine (Khib) are newly identified post-translational modifications and function as regulators of transactivation in mammals. In this study, we investigated protein post-translational Kcr and 2-hydroxyisobutyrylation in the ovarian tissues of piglets. A total of 653 overlapping proteins among differentially modified proteins were identified for both crotonylation and 2-hydroxyisobutyrylation. Gene Ontology enrichment analysis indicated that 653 DMPs were significantly enriched in nucleosome organization, chromatin assembly, DNA packaging, peptide biosynthetic process and peptide metabolic process. Kyoto Encyclopedia of Genes and Genomes (KEGG) pathway analysis showed enrichment in proteasome, ribosome, fatty acid elongation, pyruvate metabolism and pentose phosphate pathway. Fifteen DMPs were identified in the proteasome pathway, of which PSMC6 and PSMB7 were the core proteins. In addition, the significant changes in Kcr and Khib in the complex subunits of the proteasome may be involved in cell cycle processes during oocyte development. Forty-four DMPs with both Kcr and Khib modifications were related to the ribosome pathway. The regulated ribosome pathway may indicate that Kcr and Khib comodified proteins participate in protein synthesis during oocyte development. Western blot and immunofluorescence staining results supported the reliability of the sequencing results. Our results may provide a valuable resource to help illuminate the roles of Kcr and Khib in ovarian development and may serve as new tools to better control diseases.

## 1 Introduction

Ovarian function influences diverse aspects of fertility and reproductive lifespan by regulating oocyte supply and hormone secretion. In the past decade, omics—which refers to the application of high-throughput techniques—has been applied to explore ovarian physiology; numerous proteins have been found to be involved in the regulation of ovarian function throughout follicular development, oocyte maturation, ovulation and follicular atresia (Ma et al., 2022). At present, increasing evidence indicates that the functions of these proteins in regulating ovarian physiology usually involve precisely coordinated post-translational modification (PTM) (Christensen et al., 2022; Qin et al., 2022). Recently, studies reported that PTM exhibits profound effects on the female reproductive system, such as the meiosis of oocytes (Slawson and Duncan, 2015), zygote development (Zhou et al., 2019) and granulosa cell function (Wang et al., 2022). This omnipresent PTM has evident gonadal implications; however, its role in female reproductive processes, specifically ovarian folliculogenesis and endocrine function, remains largely unknown.

Recently, the important role of PTM in human and animal physiological development and pathological processes has attracted considerable attention. PTMs are enzymatic modifications of proteins that occur after translation, and these modifications mainly include succinylation, phosphorylation, methylation, acetylation, glycosylation and ubiquitination, which alter the physical or chemical properties, cellular position, function or stability of proteins (Yin et al., 2019). With the recent explosion of protein discovery, an increasing number of novel PTMs are being identified. Increasing evidence has shown that PTMs do not exist independently of other types of PTMs and that different PTMs can occur at the same site (Dong et al., 2021). Lysine crotonylation (Kcr), a novel identified PTM, was first reported in human spermatogenic cells and occurs in both core histone and nonhistone proteins in various species (Tan et al., 2011). A previous study demonstrated that changes in Kcr levels reflect the genomic stability of the cell (Fu et al., 2018). Recent research has provided data on the relationship between Kcr and oocyte development. Luo et al. (2020) first demonstrated that Kcr of H4K5 significantly accumulated in the oocytes of diabetic mice. In addition, recent studies have suggested that maternal aging can have an effect on the Kcr level, which is related to the poor quality of oocytes (Zhuan et al., 2022). Similar to Kcr, lysine 2-hydroxyisobutyryllysine (Khib) is a newly identified PTM and was first found in histone proteins from humans and mice (Dai et al., 2014). The majority of Khib proteins have been localized exclusively or partially in the nucleus and cytosol rather than mitochondria, which suggests that Khib may participate in intracellular processes and cell structure (Huang, Tang, and Dai, 2020). In terms of function, Khib can affect the catalytic activity by separating the substrate and active site (Dong et al., 2018). In addition, Khib is related to cellular glucose metabolism, which in turn affects cell survival (Huang et al., 2018). A recent study reported that the global protein expression of Khib increased significantly in mice with physiological ovarian aging (Wei et al., 2021). However, during tissue and organ development, most PTMs do not occur in isolation. Evidence for crosstalk between Kcr and Khib modifications has been found in various regulatory networks (Dong et al., 2021; Xie et al., 2022). Recent data have demonstrated that Kcr and Khib comodifications influence the number of immunocytes and further induce immune senescence in patients with end-stage renal disease through the glycolysis/gluconeogenesis pathway (Dong et al., 2021). Significant changes in Kcr and Khib in differentially modified proteins were observed in patients with systemic lupus erythematosus compared with healthy people (Xie et al., 2022). Although Kcr and Khib were observed in oocytes, it is unclear whether Kcr and Khib commodification occurs during ovarian development.

Therefore, we hypothesized that Kcr and Khib comodifications are involved in ovarian development in piglets. In this study, liquid chromatography−tandem mass spectrometry (LC−MS/MS) was performed to investigate the key features of Kcr and Khib modifications in piglet ovarian tissues.

## 2 Material and methods

### 2.1 Sample preparation

The ovaries of crossbred piglets (Duroc × Landrace × Yorkshire) at 3, 7, 15, and 30 days old (*n* = 3) were collected at a local slaughterhouse and transferred to a sterile laboratory in sterile phosphate-buffered saline (PBS) at 37°C within 1 h after slaughter. Each piglet ovary was cleared from adherent tissue and washed three times with PBS. The ovaries were mixed and ground in liquid nitrogen into cell powder and then transferred to a 5-mL centrifuge tube. Next, four volumes of lysis buffer (8 M urea, 1%, Sigma‒Aldrich, Saint Louis, United States; Protease Inhibitor Cocktail, Merck Millipore, Billerica, United States; 3 μM TSA, MedChemExpress, South Brunswick, United States and 50 mM NAM, Sigma‒Aldrich, Saint Louis, United States) were added to the cell powder, followed by sonication three times on ice using a high intensity ultrasonic processor (Scientz, Ningbo, China). The remaining debris was removed by centrifugation at 12,000 g at 4°C for 10 min. Finally, the supernatant was collected, and the protein concentration was determined with a BCA kit according to the manufacturers instructions. The intestines, lung, muscle, spleen, kidney, stomach and testicle of 7-day-old piglets were quickly collected for further analyses.

### 2.2 Trypsin digestion

For digestion, the protein solution was reduced with 5 mM dithiothreitol for 30 min at 56°C and alkylated with 11 mM iodoacetamide for 15 min at room temperature in darkness. The protein sample was then diluted by adding 100 mM TEAB to urea concentrations less than 1 M. Finally, trypsin was added at a 1:50 trypsin-to-protein mass ratio for the first digestion overnight and a 1:100 trypsin-to-protein mass ratio for a second 4 h digestion.

### 2.3 Affinity enrichment

#### 2.3.1 Pan antibody-based PTM enrichment

For enrichment of Kcr- and Khib-modified peptides, tryptic peptides dissolved in NETN buffer (100 mM NaCl, 1 mM EDTA, 50 mM Tris-HCl, 0.5% NP-40, pH 8.0) were incubated with prewashed anti-crotonyllysine antibody beads (lot number PTM-503, PTM Bio, Hangzhou, China) or anti-2-hydroxyisobutyryllysine antibody beads (lot number PTM-804, PTM Bio, PTM Bio, Hangzhou, China) at 4°C overnight with gentle shaking. Then, the beads were washed four times with NETN buffer and twice with H_2_O. The bound peptides were eluted from the beads with 0.1% trifluoroacetic acid. Finally, the eluted fractions were combined and vacuum-dried. For LC‒MS/MS analysis, the resulting peptides were desalted with C18 ZipTips (Merck Millipore, Billerica, United States) according to the manufacturers instructions.

#### 2.3.2 LC‒MS/MS analysis

The tryptic peptides were dissolved in 0.1% formic acid (solvent A) and directly loaded onto a homemade reversed-phase analytical column (15-cm length, 75 μm i.d.). The gradient comprised an increase from 6% to 23% solvent B (0.1% formic acid in 98% acetonitrile) over 26 min, 23%–35% in 8 min and climbing to 80% in 3 min then holding at 80% for the last 3 min, all at a constant flow rate of 400 nL/min on an EASY-nLC 1000 UPLC system (Thermo Fisher Scientific, Waltham, United States).

The peptides were subjected to an NSI source followed by tandem mass spectrometry (MS/MS) in Q ExactiveTM Plus (Thermo Fisher Scientific, Waltham, United States) coupled online to the UPLC. The electrospray voltage applied was 2.0 kV. The m/z scan range was 350–1800 for the full scan, and intact peptides were detected in the Orbitrap at a resolution of 70,000. Peptides were then selected for MS/MS using an NCE setting of 28, and the fragments were detected in the Orbitrap at a resolution of 17,500. A data-dependent procedure alternated between one MS scan followed by 20 MS/MS scans with 15.0 s dynamic exclusion. Automatic gain control (AGC) was set at 5E4. The fixed first mass was set as 100 m/z. The mass spectrometry proteomics data have been deposited to the ProteomeXchange Consortium (http://proteomecentral.proteomexchange.org) via the iProX partner repository with the dataset identifier PXD038469.

#### 2.3.3 Database search

The resulting MS/MS data were processed using the MaxQuant search engine (v.1.5.2.8). Tandem mass spectra were searched against the UniProtKB *Sus scrofa* database (Taxon ID 9823, 20180506 downloaded from UniProt database, database sequence number: 40710) concatenated with the reverse decoy database. Trypsin/P was specified as the cleavage enzyme allowing up to 4 missing cleavages. The mass tolerance for precursor ions was set as 20 ppm in the first search and 5 ppm in the main search, and the mass tolerance for fragment ions was set as 0.02 Da. For identification of crotonylation, carbamidomethyl on Cys was specified as a fixed modification, and lysine (Lys) crotonylation modification and oxidation on Met were specified as variable modifications. For identification of 2-hydroxyisobutylation, carbamidomethyl on Cys was specified as a fixed modification, and Lys 2-hydroxyisobutylation modification and oxidation on Met were specified as variable modifications. The false discovery rate (FDR) was adjusted to <1%, and the minimum score for modified peptides was set to >40.

#### 2.3.4 Immunoprecipitation (IP) assay

Proteins were lysed in lysis buffer consisting of a protease inhibitor cocktail. IP was carried out by incubating with or without 25 μL of anti-VCP (Cat#: PTM-5823, PTM Bio, Hangzhou, China). Thirty microliters of Protein A/G PLUS-Agarose (Cat#: sc-2003, Santa Cruz Biotech, Santa Cruz, CA) was added to the solution and incubated for 6 h on ice. After incubation, we washed the bound proteins four times with PBS buffer and then suspended them in 40 μL of PBS buffer. Proteins in PBS buffer were analyzed by sodium dodecyl sulfate‒polyacrylamide gel electrophoresis (SDS‒PAGE) and Western blotting.

### 2.4 Western blotting

Thirty micrograms of total protein was loaded into each well of a 12% SDS–PAGE gel, and the proteins were separated by electrophoresis. The proteins were then transferred to PVDF membranes (Millipore; Bedford, MA, United States). After blocking, the membranes were incubated with anti-crotonyl-histone H2B (Lys20) (Cat#: PTM-512, PTM Bio, Hangzhou, China) (1: 2000), anti-crotonyl-histone H2B (Lys34) (Cat#: PTM-514, PTM Bio, Hangzhou, China) (1: 1000), anti-crotonyllysine (Cat#: PTM-502, PTM Bio, Hangzhou, China) (1: 200), anti-2-hydroxyisobutyryl-histone H3 (Lys14) (Cat#: PTM-881, PTM Bio, Hangzhou, China) (1: 1000), anti-2-hydroxyisobutyryl-histone H3 (Lys79) (Cat#: PTM-845, PTM Bio, Hangzhou, China) (1: 1000), anti-2-hydroxyisobutyryl-histone H4 (Lys5/8/12) (Cat#: PTM-850, PTM Bio, Hangzhou, China) (1: 1000), anti-GAPDH (Cat#: PTM-6620, PTM Bio, Hangzhou, China) (1: 2000) and anti-VCP (Cat#: PTM-5823, PTM Bio, Hangzhou, China) (1: 2000) overnight at 4°C. Then, the membranes were incubated with HRP-labeled secondary antibodies. Finally, the protein bands were visualized using Image-Pro Plus 6.0 software (Media Cybernetics, Inc., Silver Spring, MD, United States) and measured with Quantity One software (Bio-Rad Laboratories, Hercules, CA, United States).

### 2.5 Immunofluorescence staining

The tissues were fixed overnight and embedded in paraffin. After hydration and permeabilization, 3% H_2_O_2_ was used to remove endogenous peroxidase. For permeabilization, 0.1% Triton X-100 was gently dripped onto the tissue section and incubated at 37°C for 20 min. Sodium citrate antigen retrieval solution was used for antigen retrieval, and 10% goat serum was used for blocking. For antigen retrieval, 1 L of sodium citrate antigen retrieval solution was heated to boiling in a pressure cooker. The sections were placed in the pressure cooker and heated at high (2100 W) until steam came out of the top, and then, the pressure cooker was turned down to low heat (800 W) for 2.5 min. Goat serum was then incubated with the slides for blocking. The slides of tissues were incubated with primary antibodies at 37°C for 2 h, including crotonyllysine (Cat#: PTM-502, PTM Bio, Hangzhou, China) (1: 200) and 2-hydroxybutyryllysine (Cat#: PTM-801, PTM Bio, Hangzhou, China) (1: 200). The nuclei were washed three times with PBS and incubated for 1 h at room temperature in a 1:500 dilution mixture of Alexa-labeled secondary antibodies (Invitrogen, Life Technologies Corp, Carlsbad, CA, United States) at 37°C for 2 h. Then, the nuclei were counterstained with DAPI (4,6-diamidino-2-phenylindole). Then, slides were observed by a fluorescence microscope (Nikon, Inc., Melville, NY, United States).

### 2.6 Bioinformatics analysis

Proteins with a change in the differential folding value greater than 1.5 times or less than 1/1.5 were defined as DMPs. DMPs dually modified by Kcr and Khib were screened by a Venn diagram (https://bioinfogp.cnb.csic.es/tools/venny/index.html), and WOLF-PSORT was used to classify subcellular localization. The function and features of DMPs dually modified by Kcr and Khib were annotated by Gene Ontology (GO) enrichment analysis. The interaction was annotated by Kyoto Encyclopedia of Genes and Genomes (KEGG) enrichment analysis. STRING and Cytoscape v3.9.0 were used to construct protein-protein interaction (PPI) networks. Core genes were identified by the cytoHubba plugin. A corrected *p*-value <0.05 was considered statistically significant in all bioinformatics analyses.

## 3 Results

### 3.1 Protein identification

**FIGURE 1:**
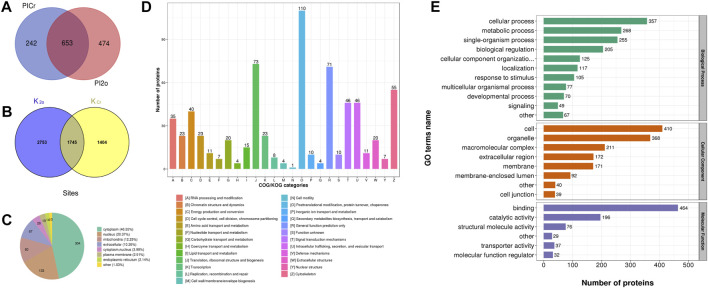
Qualitative analysis of lysine crotonylation and 2-hydroxyisobutyrylation in the ovarian tissue mixtures of piglets at 3, 7, 15, and 30 days old. **(A)** Venn diagram showing the overlapping proteins modified by dual lysine crotonylation and 2-hydroxyisobutyrylation. **(B)** Venn diagram showing the overlapping modified sites between lysine crotonylation and 2-hydroxyisobutyrylation. **(C)** Subcellular location of the overlapping DMPs of dual Kcr and Khib. **(D)** Clusters of orthologous groups of protein/EuKaryotic orthologous groups (COG/KOG) functional classification analysis. **(E)** Functional category of the overlapping DMPs of dual lysine crotonylation and 2-hydroxyisobutyrylation in GO terms.

In this study, Kcr and Khib were systematically identified in the ovarian tissue mixture of piglets aged 3, 7, 15 and 30 days to investigate the potential role of Kcr and Khib modifications in the ovarian development of piglets. A total of 20950 secondary spectra were obtained by mass spectrometry of the Kcr modification. After the library of protein theoretical data was searched, the available effective number of secondary spectra of mass spectrometry was 5622, and the utilization rate of the spectrum was 26.8% (Table 1). A total of 3149 crotonylation sites on 895 proteins were identified in mixed ovaries (Supplementary Table S1). Information on the Kcr-modified peptide is shown in Supplementary Table S1. In addition, a total of 21839 secondary spectra were obtained by mass spectrometry of the Khib modification. After the protein theoretical data database was searched, the available effective number of the secondary spectrum of mass spectrometry was 7268, and the utilization rate of the spectrum was 33.3% (Table 1). A total of 4498 sites on 1127 proteins were identified by 2-hydroxyisobutyrylation proteomics analysis in mixed ovaries (Supplementary Table S2). Information on the Khib-modified peptide is shown in Supplementary Table S2. Generally, compared to Kcr, the Khib modification was more abundant in the ovaries of piglets. A Venn diagram showed that 653 overlapping proteins among DMPs were comodified by Kcr and Khib (Figure 1A and Supplementary Table S3). The proportion of DMPs that were directly modified by Kcr and Khib was 72.9% in Kcr DMPs and 57.9% in Khib DMPs. Then, we sorted out 1745 overlapping sites among modified sites of both Kcr and Khib modifications (Figure 1B and Supplementary Table S4).

**TABLE 1:** Basic statistical table of MS results.

Total spectrum	Matched spectrum	Peptides	Modified peptides	Identified proteins	Identified sites
20950	5622	4077	3128	895	3149
21839	7268	4961	4459	1127	4498

### 3.2 Features of the DMPs dually modified by Kcr and Khib

The subcellular localization of DMPs dually modified by Kcr and Khib was distributed in the cytoplasm (46.55%), nucleus (20.37%), mitochondria (12.25%), extracellular (10.26%) cytoplasm, nucleus (3.98%), plasma membrane (2.91%) and endoplasmic reticulum (2.14%) (Figure 1C and Supplementary Table S5). Clusters of Orthologous Groups of protein/EuKaryotic Orthologous Groups (COG/KOG) analysis was also used to predict the functional classification of dually modified DMPs. In all functional ontologies, the largest category was “posttranslational modification, protein turnover, chaperones” (110). The “translation, ribosomal structure, and biogenesis” (73) and “general function prediction only” (71) groups contained relatively more proteins, followed by “cytoskeleton” (55), “signal transduction mechanisms” (46) and “intracellular trafficking, secretion, and vesicular transport” (46) (Figure 1D and Supplementary Table S6).

Based on the GO annotation, functional classification was performed to primarily investigate the functions of 653 DMPs dually modified by Kcr and Khib in the ovaries of piglets. The biological process in the GO enrichment analysis showed significant enrichment of cellular process, metabolic process, single-organism process and biological regulation (Figure 1E and Supplementary Table S7). In the cellular component category, these proteins were highly enriched in cell, organelle, macromolecular complex and extracellular region (Figure 1E and Supplementary Table S7). At the molecular function level, the majority of DMPs dually modified by Kcr and Khib were involved in binding, catalytic activity, structural molecule activity and transporter activity (Figure 1E and Supplementary Table S7).

### 3.3 Functional enrichment of overlapping DMPs of dual Kcr and Khib

**FIGURE 2:**
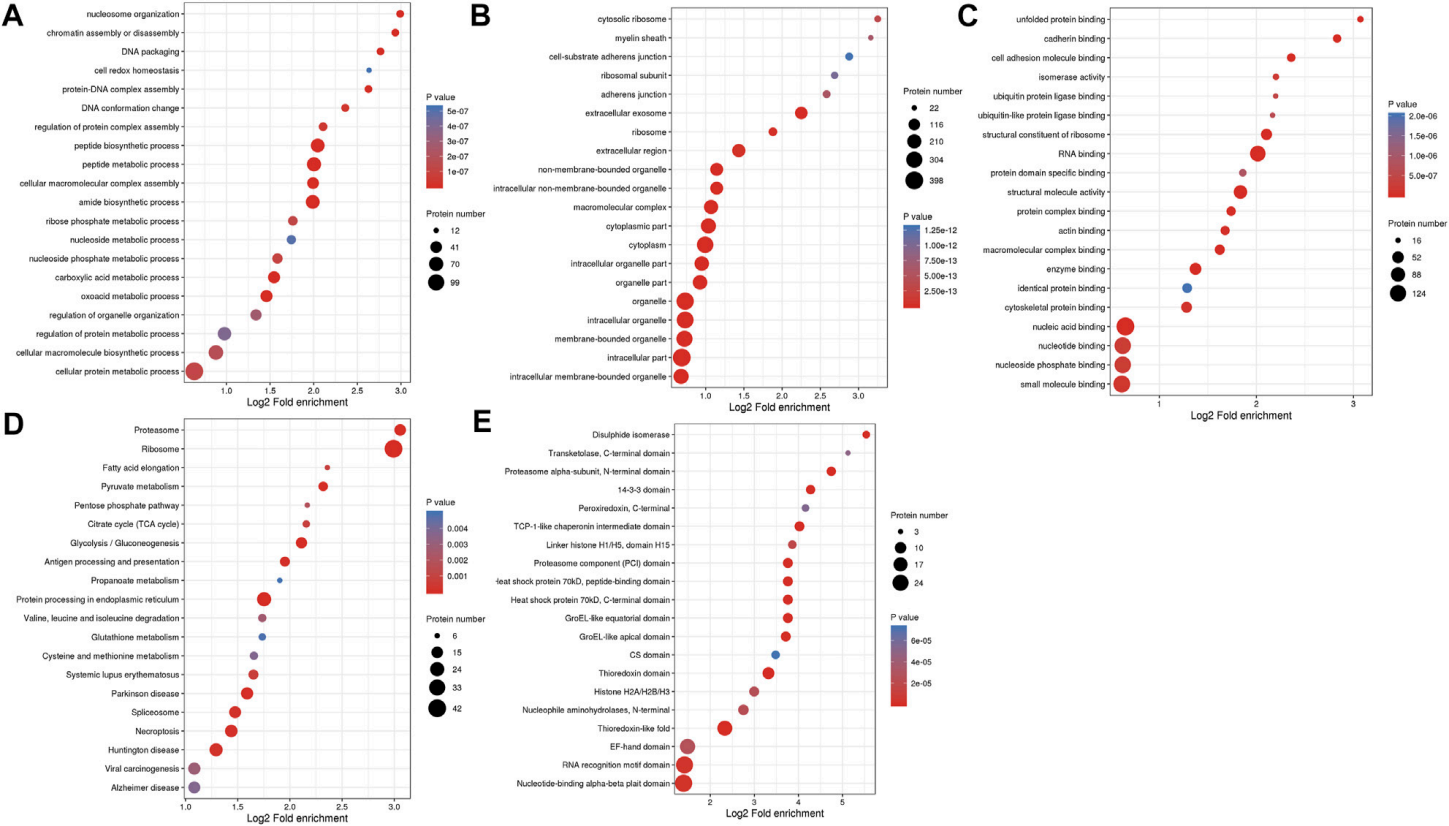
GO enrichment analysis and KEGG pathway enrichment analysis of overlapping DMPs of dual Kcr and Khib. **(A)** Biological process in the GO enrichment analysis. **(B)** Cellular component in the GO enrichment analysis. **(C)** Molecular function in the GO enrichment analysis. **(D)** KEGG analysis of overlapping DMPs of dual Kcr and Khib. **(E)** The protein domain analysis of overlap DMPs of dual Kcr and Khib.

To further demonstrate the role and function of DMPs dually modified by Kcr and Khib in ovarian development, we performed GO and Kyoto Encyclopedia of Genes and Genomes (KEGG) pathway enrichment analyses. According to biological process enrichment classification, nucleosome organization, chromatin assembly, DNA packaging, peptide biosynthetic process and peptide metabolic process were significantly enriched (Figure 2A and Supplementary Table S8). At the cellular component level, these proteins were highly enriched in cytosolic ribosomes, myelin sheaths, cell-substrate adherens junctions, adherens junctions and extracellular exosomes (Figure 2B and Supplementary Table S8). The overlapping DMPs in the molecular function category were highly enriched in unfolded protein binding, cadherin binding, cell adhesion molecule binding, isomerase activity and ubiquitin protein ligase binding (Figure 2C and Supplementary Table S8).

In the KEGG pathway analysis, the proteasome was the top significantly enriched pathway. In addition, ribosome, fatty acid elongation, pyruvate metabolism and pentose phosphate pathway were enriched (Figure 2D and Supplementary Table S9). Furthermore, protein domain enrichment analysis identified 20 significantly enriched domains, including the disulfide isomerase, transketolase, C-terminal domain and proteasome alpha-subunit, N-terminal domain (Figure 2E and Supplementary Table S10).

### 3.4 Characteristics of the proteasome pathway

**FIGURE 3:**
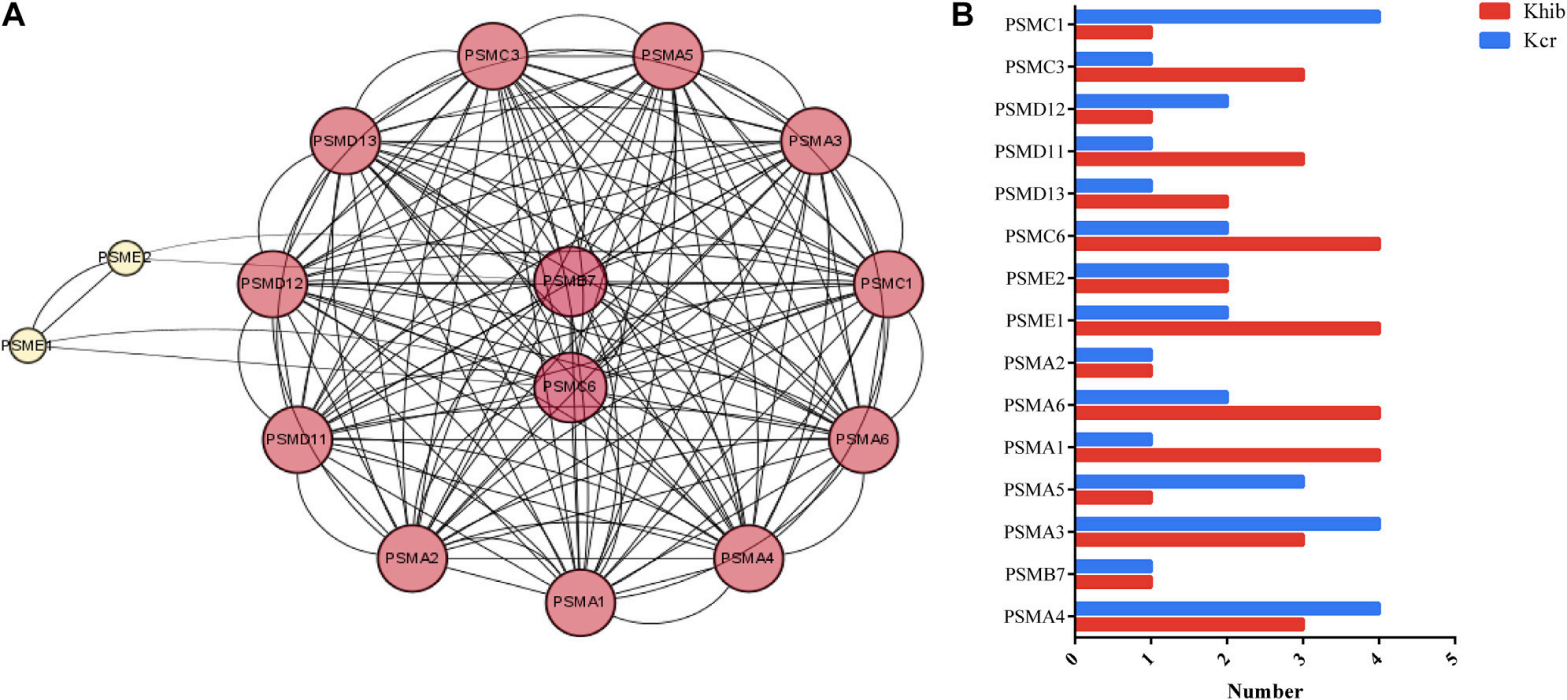
DMPs dually modified by Kcr and Khib in the proteasome pathway. **(A)** PPI network of the DMP-enriched proteasome pathway. **(B)** Numbers of modified sites of Kcr and Khib in the proteasome pathway.

The proteasome process was a highly enriched pathway in KEGG analysis and is closely associated with the development of ovaries. Fifteen nodes, including PSMA4, PSMB7, PSMA3, PSMA5, PSMA1, PSMA6, PSMA2, PSME1, PSME2 and PSMC6, and 81 interactions were involved in the network (Figure 3A). PSMC6 and PSMB7 were the top 2 hub genes. PSMC6 encodes one of the ATPase subunits, a member of the triple-A family of ATPases that exhibits chaperone-like activity. Two Kcr sites and four Khib sites were identified in PSMC6. Two sites (K34 and K220) in PSMC6 were identified as possessing both Kcr and Khib modifications (Figure 3B). In addition, 18 sites (K13, K34, K 35, K50, K61, K65, K72, K103, K127, and K156) were comodified by both Kcr and Khib in the fifteen nodes.

### 3.5 Characteristics of the ribosome pathway

**FIGURE 4:**
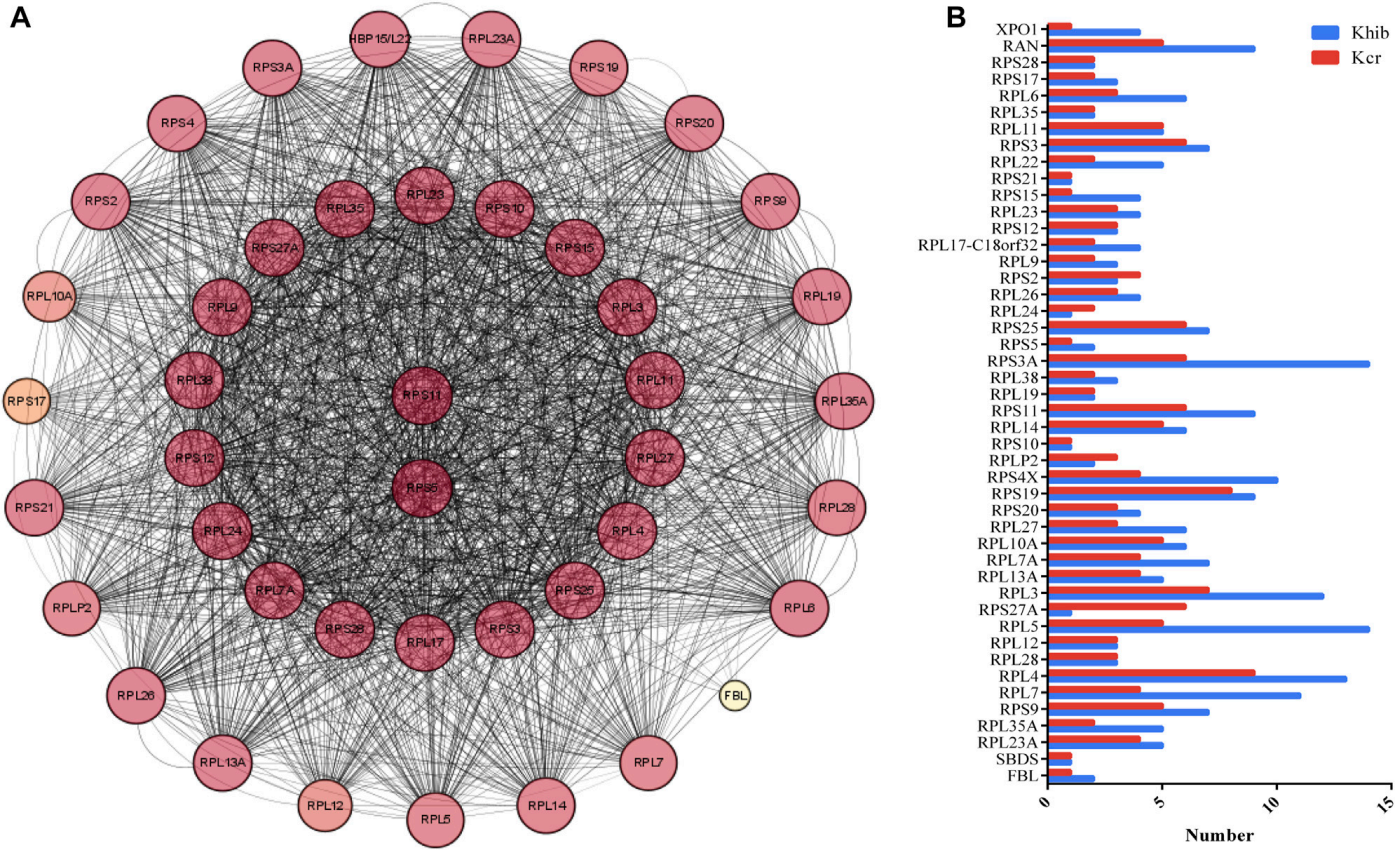
DMPs dually modified by Kcr and Khib in the ribosome pathway. **(A)** PPI network of DMP-enriched ribosome pathways. **(B)** Numbers of modified sites of Kcr and Khib in the ribosome pathway.

Forty-four nodes, including RPS11, RPS5, RPL7, RPL4, RPL5, RPS4X, RPS3A, RPS3, and RAN, and 834 interactions were involved in the network (Figure 4A). RPS11 and RPS5 were the top 2 hub genes. RPS11 encodes a member of the S17P family of ribosomal proteins, that is, a component of the 40S subunit and is cotranscribed with the small nucleolar RNA gene U35B, which is located in the third intron. Lysine K20, K48, K79, K144, K45, and K30 in RPS11 co-occur with Kcr and Khib at the same site. There are 95 sites (K15, K155, K93, K116, K66, K75, and K31) comodified by both Kcr and Khib (Figure 4B).

### 3.6 Validation of Kcr and Khib modifications using Western blotting and immunofluorescence

**FIGURE 5:**
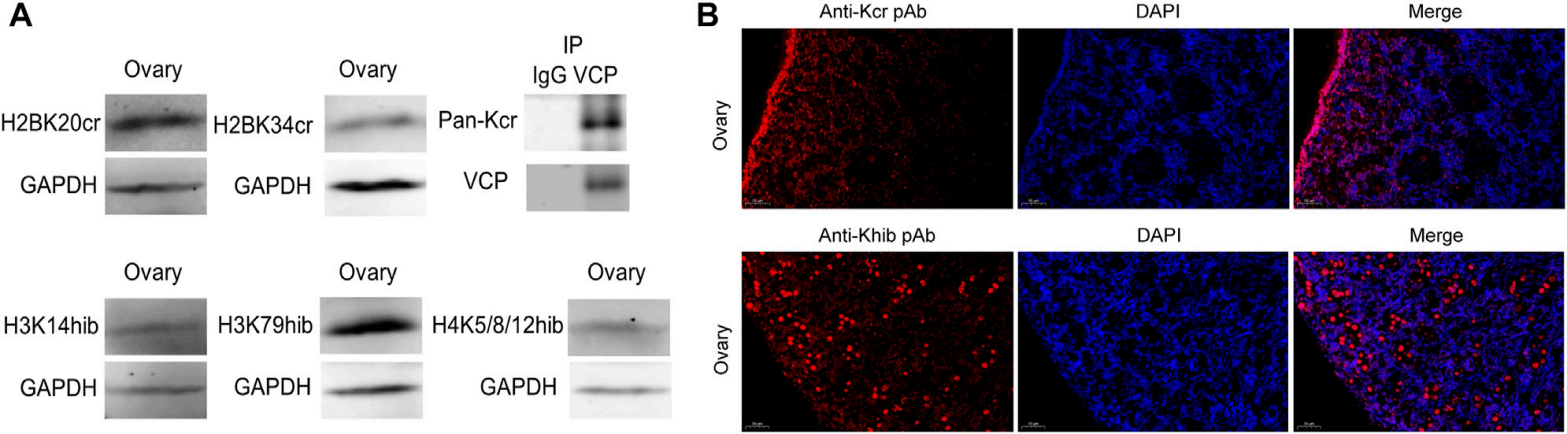
Western blotting and immunofluorescence analysis of Kcr and Khib in the ovarian tissue mixture of piglets. **(A)** Validation of sequencing results in the ovarian tissue mixture of piglets at 3, 7, 15 and 30 days old by Western blot analysis. **(B)** Immunofluorescence staining of the ovarian tissue of piglets at 7 days old with the anti-Kcr antibody or the anti-Khib antibody.

**FIGURE 6:**
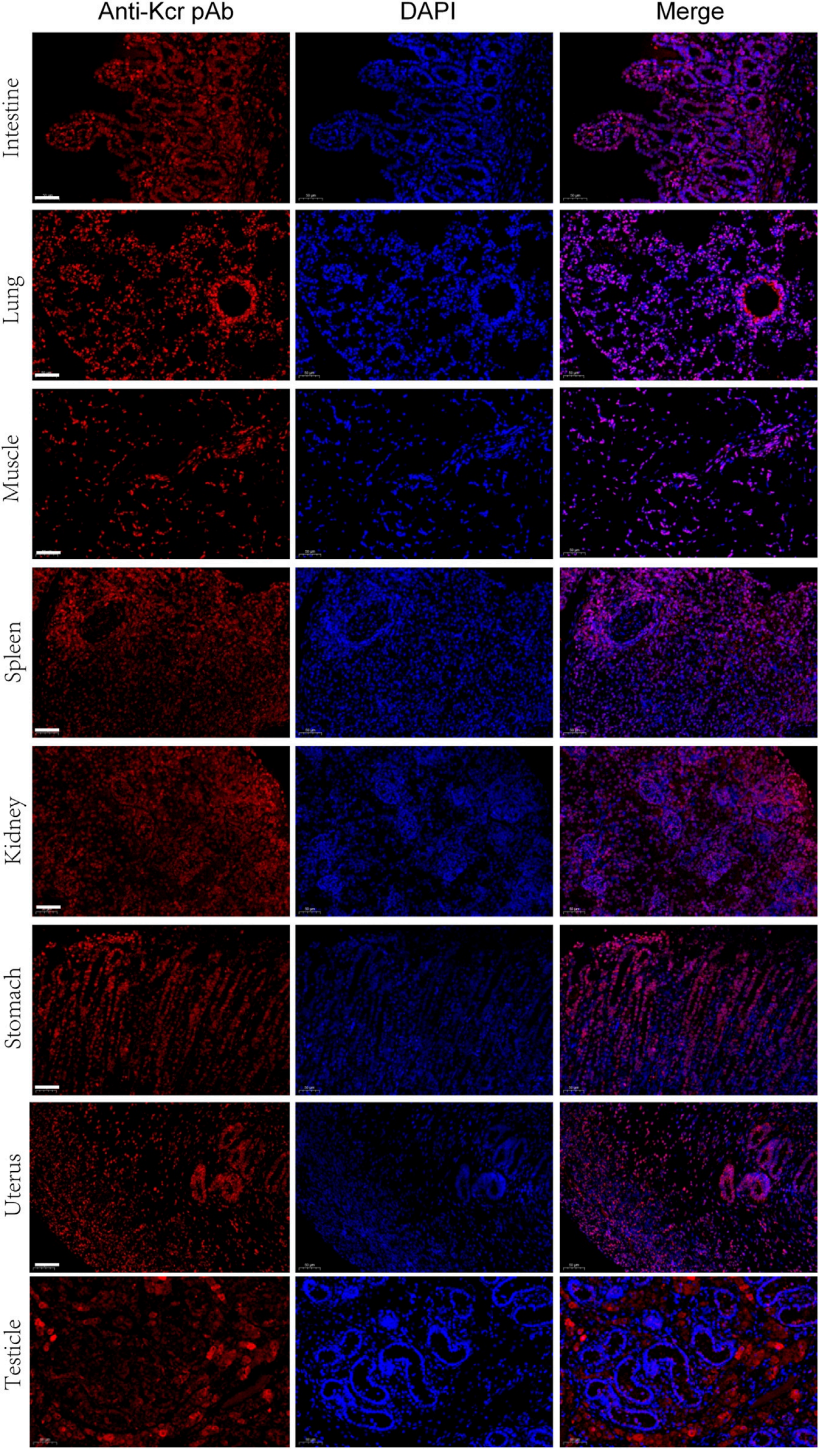
Immunofluorescence staining of the tissues of piglets at 7 days old with the anti-Kcr antibody (red). Nuclei were stained with DAPI (blue). Scale bar = 50 μm.

**FIGURE 7:**
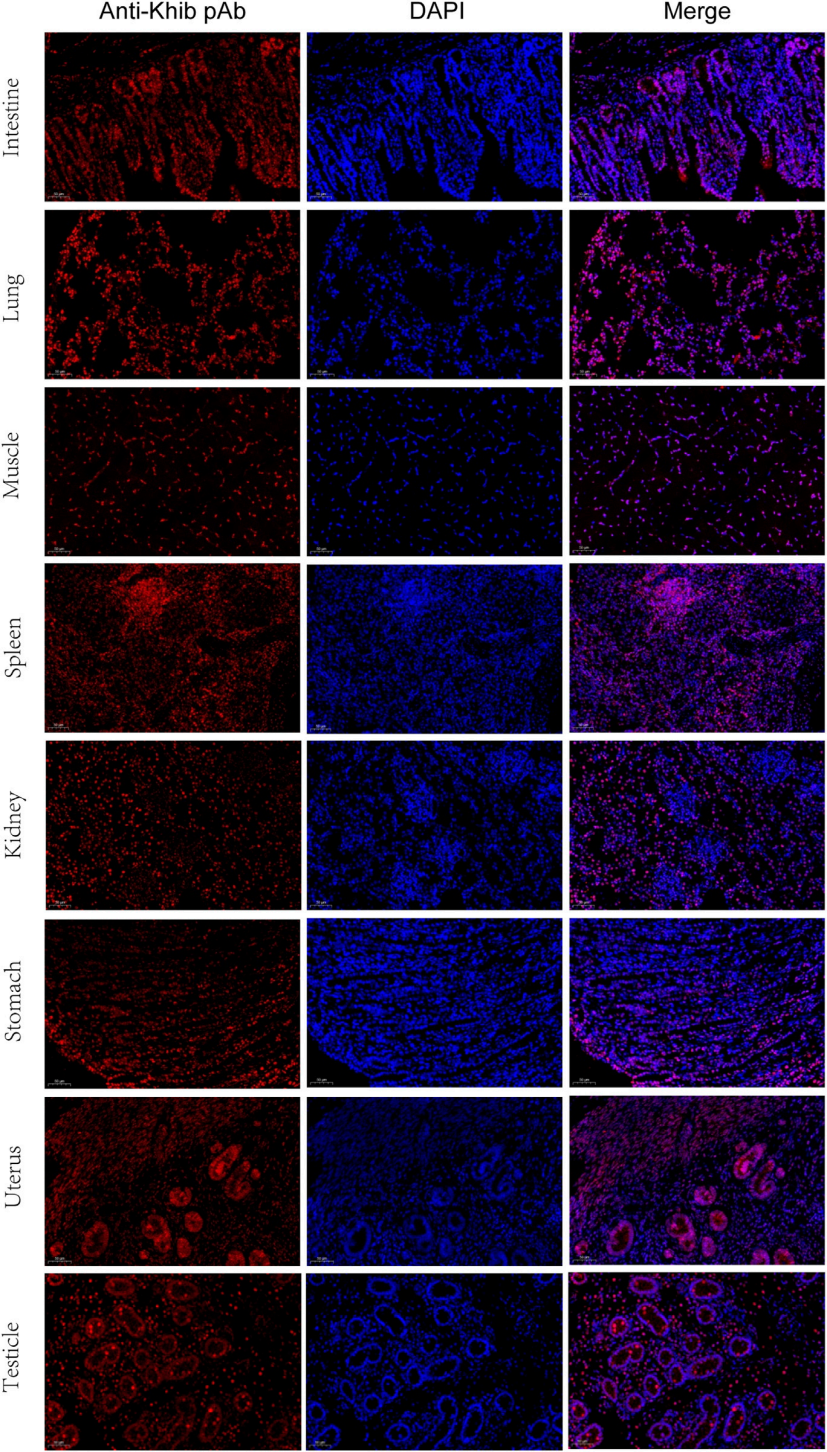
Immunofluorescence staining of the tissues of piglets at 7 days old with the anti-Khib antibody (red). Nuclei were stained with DAPI (blue). Scale bar = 50 μm.

For further validation of the above results, Kcr- and Khib-related antibodies were selected and analyzed by Western blotting and immunofluorescence. The results showed that Kcr modification at the H2BK20 and H2BK34 sites was detected in ovarian tissue (Figure 5A). Valosin-containing protein (VCP), a Kcr-modified protein, was immunoprecipitated by an anti-VCP antibody, in which Kcr was clearly present (Figure 5A). Then, immunofluorescence staining was performed to visualize the cellular localization of Kcr-modified proteins. The results revealed that Kcr-modified proteins were localized in the somatic cells and epithelium of ovaries (Figure 5B). In addition, the Khib modification of H3K14, H3K79 and H4K5/8/12 was confirmed by Western blotting in ovarian tissue (Figure 5A). In contrast to the location of Kcr-modified proteins, the expression of Khib-modified proteins was mainly expressed in oocytes (Figure 5B). In addition, the distribution of Kcr- and Khib-modified proteins was visualized in the intestines, lung, muscle, spleen, kidney, stomach, uterus and testicle in 7-day-old piglets (Figure 6; Figure 7).

## 4 Discussion

Kcr and Khib modifications are two new types of protein acylation modifications. Research on these two modifications has been extended from histone modification to nonhistone modification in various organisms from humans to plants (Tan et al., 2011; Xu et al., 2017). However, information about Kcr and Khib modifications in mammalian ovarian development is extremely limited. In this study, we identified 653 DMPs dually modified by Kcr and Khib in piglet ovary mixtures from 3 to 30 days old. The results of coimmunoprecipitation, Western blotting and immunofluorescence confirmed the LC–MS/MS data. KEGG analysis showed enrichment in the proteasome, ribosome, fatty acid elongation, pyruvate metabolism and pentose phosphate pathway.

During the past few years, more evidence has accumulated to suggest that the proteasome pathway plays a crucial role in regulating oocyte meiotic maturation, chromosome segregation and polar body extrusion in mammals (Karabinova, Kubelka, and Susor, 2011). Protein degradation through the proteasome pathway is tightly regulated by a variety of proteins to prevent random protein degradation. At the core of the proteasome is the 26S proteasome, a multisubunit proteolytic complex consisting of a central catalytic 20S core particle and a 19S regulatory particle (Aharoni et al., 2022). This regulatory particle binds covalently to a polyubiquitin chain on a protein substrate specifically designed for degradation, thereby specifically controlling the proteolytic function of the proteasome. A previous study demonstrated that the proteasome pathway regulates the progression of oocyte meiosis by influencing the degradation of RAB7 (Jin et al., 2022). This finding was similar to those in the oocyte of proteasome disruption, which presented chromosome compaction accompanied by chromosome segregation failure and arrest at the MI stage (Jin et al., 2019). Woodman et al. (2022) demonstrated that deficiency in the proteasome pathway altered folliculogenesis and the morphology of oocytes and disrupted estrous cyclicity in mice. In our study, abundant Kcr and Khib modification sites were identified in the complex subunits of the 20S core and the 19S regulator of the 26S proteasome, such as PSMB7, PSMC6, PSMA1, PSMC1, and PSME1. Previous studies reported that the complex subunits of the proteasome were involved in meiotic and cell cycle progression (Gómez-H et al., 2019; Liu et al., 2019). These findings indicate that the significant changes in Kcr and Khib in the complex subunits of the proteasome may participate in cell cycle processes by regulating protein degradation during oocyte development.

Oogenesis is an energy-consuming process that requires a large amount of protein and RNA, so ribosomes are in high demand during oocyte maturation (Breznak, Kotb, and Rangan, 2023). The main function of the ribosome is to translate messenger RNA into proteins in the nucleolus (Sun et al., 2022). The ribosome is composed of a small (40S) and a large (60S) subunit in eukaryotic cells. The small subunit of the ribosome is anchored to the mRNA so that a set of three nucleotides (a codon) can be presented to a specific tRNA at the amino-acyl site (A site) that carries the amino acid. The large subunit of the ribosome links each amino acid to synthesize the polypeptide chain at the peptide-base site (the P site), while the empty tRNA is ejected from the ribosome at the exit site (the E site) (Liu et al., 2022). In our study, the 44 DMPs with both Kcr and Khib modifications are related to the ribosome pathway, which is involved in the two key components of ribosomes (40S and 60 subunits). Previous studies have demonstrated that alterations in functional ribosomal genes can lead to tissue-specific defects and developmental disorders (Paolini et al., 2017). Paolini et al. (2017) reported that mutations in the RPS23 gene reduce the stability of the US12 protein and affect its ability to correctly decode mRNA, resulting in malformation in children. A recent study found that altering the ribosome pathway may affect the number of healthy oocytes and oocyte maturation (Nakanishi et al., 2022). Another study found that bovine follicular fluid contained numerous ribosome proteins, which may serve to compact different RNAs to regulate gene expression and RNA degradation and might transfer ribosomal constituents to the oocyte (Uzbekova et al., 2020). In addition, sufficient ribosomes are essential for sustaining the required levels of protein synthesis during oogenesis (Talbot et al., 2023). Thus, we propose that Kcr and Khib modifications may influence oocyte development through the ribosome pathway by regulating protein synthesis.

We provided evidence that Kcr modification at the H2BK20 and H2BK34 sites and Khib modification at H3K14, H3K79 and H4K5/8/12 occur in neonatal porcine ovaries. However, according to the immunofluorescence staining results, the mechanisms of Kcr and Khib involvement in ovarian development may be different. The unique expression of Kcr-modified proteins in somatic cells suggests that these proteins may play an important role in somatic cell‒oocyte communication, leading to the formation of early granulosa cells. In contrast to Kcr-modified protein, positive Khib signals in the oocytes of primordial follicles on 7-day-old piglets suggested that those proteins may play a critical role in folliculogenesis. More work is needed to elucidate the potential functions of Kcr and Khib modifications on follicle morphogenesis and folliculogenesis. Interestingly, the distribution of Kcr- and Khib-modified proteins in various organs suggests that Kcr and Khib may play a potential role in organ maturation, and further research is needed.

Collectively, our quantitative proteomic analysis explored proteins dually modified with Kcr and Khib in piglet ovaries from 3 to 30 days old. To our knowledge, this is the first comprehensive analysis of Kcr and Khib dual modifications in the ovaries of mammals. The overlapping DMPs of dual Kcr and Khib were enriched in the proteasome and ribosome pathways. Our work may provide a valuable resource to help illuminate the roles of Kcr and Khib in ovarian development and may serve as new tools to better control diseases.

## Data Availability

The datasets presented in this study can be found in online repositories. The names of the repository/repositories and accession number(s) can be found below: http://www.proteomexchange.org/, PXD038469.
