# Supplementary material for: Qualitative lysine crotonylation and 2-hydroxyisobutyrylation analysis in the ovarian tissue proteome of piglets
Source: Front Cell Dev Biol. 2023 May 15;11:1176212. doi: 10.3389/fcell.2023.1176212 (PMC10225730; doi:10.3389/fcell.2023.1176212)
Supplement: Supplementary file 5 [file Table2.DOCX]

**Table 2. Basic statistical table of MS results**

| **Total spectrum** | **Matched spectrum** | **Peptides** | **Modified peptides** | **Identified proteins** | **Identified sites** |
| --- | --- | --- | --- | --- | --- |
| 21839 | 7268 | 4961 | 4459 | 1127 | 4498 |
